# Gender-specific association of multiple risk factors with neonatal moderate or severe hypoxic ischemic encephalopathy: a cross-sectional study

**DOI:** 10.1186/s13052-024-01748-0

**Published:** 2024-09-09

**Authors:** Yiran Wang, Yaodong Zhang, Shuying Luo, Kaijuan Wang

**Affiliations:** 1 Henan Key Laboratory of Children’s Genetics and Metabolic Diseases, Children’s Hospital Affiliated to Zhengzhou University, Henan Children’s Hospital, Zhengzhou Children’s Hospital, Zhengzhou, Henan Province China; 2 Department of Epidemiology and Health Statistics, College of Public Health, Zhengzhou University, Zhengzhou, Henan Province China

**Keywords:** Hypoxic ischemic encephalopathy, Risk factors, Cross-sectional study, Gender

## Abstract

**Background:**

Neonatal hypoxic ischemic encephalopathy (HIE) leads to different degrees of neurological sequelae. The incidence of HIE is relatively high, and the causal pathways leading to HIE are still controversial. This study aimed to investigate the risk factors associated with HIE comparing differences between genders.

**Methods:**

A cross-sectional study of 196 neonates diagnosed with HIE was conducted. Based on the severity of clinical findings, HIE was classified as mild, moderate or severe. For mild HIE, the outcomes were relatively less severe, whereas moderate to severe HIE could suffer serious consequences, including death, cerebral palsy, epilepsy. T-test, chi-square test and logistic regression were used to analyze data.

**Results:**

Among the 196 neonatal HIE, 39 (19.9%) had mild HIE,157 (80.1%) had moderate or severe HIE. The logistic regression analysis showed that gender was a specific stratified characteristic of moderate or severe HIE. In the male neonates group, emergency cesarean section, abnormal labor stage and amniotic fluid contamination were associated with an increased risk of moderate or severe HIE, where the adjusted odds ratios (ORs) were 4.378 (95% confidence intervals (CI):2.263–6.382), 2.827 (95% CI:1.743–5.196) and 2.653 (95%CI:1.645–3.972), respectively. As expected, a significant additive effect was found in the interactions between emergency cesarean section and abnormal labor stage, as well as between emergency cesarean section and amniotic fluid contamination, where the relative excess risk of interaction was 2.315(95%CI:1.573–3.652) and 1.896(95%CI: 1.337–3.861) respectively.

**Conclusion:**

Emergency cesarean section, abnormal labor stage and amniotic fluid contamination were risk factors of moderate or severe HIE in neonates, and the associations were significantly correlated with male gender. Notably, coinciding incidences of emergency cesarean section with abnormal labor stage, or emergency cesarean section with amniotic fluid contamination were possibly synergistic in increasing the risk of moderate or severe HIE. These findings may assist clinicians in strengthening their awareness on risks affecting HIE and help reduce the incidence of moderate or severe HIE in clinical practice.

## Introduction

 Neonatal hypoxic ischemic encephalopathy (HIE) refers to various degrees of brain damage due to asphyxia caused by partial or total lack of oxygen. HIE is a common neonatal disease of the central nervous system that is caused by the reduction or suspension of cerebral blood flow during the perinatal period [1–5].HIE is a critical cause of neonatal death or can lead to different degrees of neurological sequelae in neonates. At present, it is difficult to evaluate the scope and severity of brain injury caused by HIE during the early diagnostic path [6–8]. There are also several major scoring systems for clinical evaluation of HIE. The Apgar score was first introduced in 1952 by an American anesthesiologist named Virginia Apgar, and it is a widely utilized tool for neonates to describe an infant’s condition after birth [9]. Based on the neurological symptoms of the neonates, HIE can be divided into three categories: mild, moderate and severe. The degree of HIE closely correlates to the risk of future neurological impairment. For neonates with mild HIE, the outcomes are relatively less severe, whereas neonates with moderate to severe HIE can suffer serious consequences, including death, cerebral palsy, epilepsy, and cognitive impairment [10, 11]. Despite significant improvements in neonatal therapy, HIE continues to exist in high income countries and remains a disproportionately high burden in low and middle income countries [12–14]. Globally, the incidence of HIE in full-term live neonates ranges from 1 to 8 per 1000 live births, and even in high income countries the incidence is 1.5 to 2 per 1000 live births [15]. Around 400,000 neonates develop neuro-developmental disorders caused by HIE worldwide each year [16]. In China, about 100,000 neonates are affected by HIE [17].

The causal pathways leading to moderate or severe HIE are complex. Previous epidemiological studies have identified several risk factors that are associated with moderate or severe HIE, including birth weight, gestational age, viral infection, placenta previa, drugs during pregnancy, fetal distress, abnormal labor stage, low Apgar score, amniotic fluid contamination, maternal obesity, and mode of delivery [18–20].However, there have been inconsistent results in studies examining the risk factors for moderate or severe HIE, which may be related to the differences in study design, study population or diagnostic criteria. Currently, the risk factors associated with moderate or severe HIE in the medical institutions in Henan province, China, are unclear. Regional research is necessary in order to establish a representative study population and to provide evidence as justification for the design of preventive strategies against moderate or severe HIE in Henan’s hospitals. In current literature, there is limited information on how gender can be a risk factor for moderate or severe HIE. Thus, we conducted a cross-sectional study to analyze conditions related with neonatal moderate or severe HIE treated in Henan Children’s Hospital, using a strict inclusion criteria based on gender-specific differences. Additionally, we also used an additive model to analyze possible interactions among the risk factors, thus providing a basis for the primary prevention of moderate or severe HIE in early clinical stage.

## Methods

### Study population

In this cross-sectional study, a total of 196 neonates diagnosed with HIE admitted to Henan Children’s Hospital from January 2014 to June 2021 were selected as the study population. Neonates were diagnosed as HIE according to” the Diagnosis of Hypoxic-ischemic Encephalopathy in Term Infants” [21]. The diagnostic criteria of HIE of the neonatal group of the Pediatric Society of Chinese Medical association were as follows: (1) there is a clear history of abnormal obstetrics that can lead to fetal distress, as well as severe fetal distress manifestations (fetal heart rate < 100 beats/min, lasting more than 5 min; and/or amniotic fluid level III contamination), or a significant history of asphyxia during delivery; among these, the amniotic fluid III contamination refers to the more viscous and turbid nature of the amniotic fluid, which has a greater impact on the fetus’s breathing, and in severe cases, it is easy to cause intrauterine hypoxia, so that the fetus is suffocated; (2) severe asphyxia at birth, including Apgar score ≤ 3 at 1 min and ≤ 5 at 5 min, and/or umbilical cord artery acidemia(pH < 7); (3) neurological symptoms appear shortly after birth and last for more than 24 h, such as changes in consciousness (hyperexcitability, lethargy, coma), changes in muscle tone (increase or decrease), abnormal primitive reflexes (weakening or disappearance of sucking and hugging reflexes), seizures in severe cases, brain stem signs (changes in breathing rhythm, pupil changes, delayed response to light) and increased bregmatic tension; (4) excluding convulsions caused by electrolyte imbalances (hypocalcemia, hypoglycemia, etc.), birth trauma and intracranial hemorrhage, as well as brain damage caused by intrauterine infections, inherited metabolic diseases, and other congenital conditions. Of the selected neonatal HIE patients, 39 neonates were diagnosed with mild HIE and 157 neonates were diagnosed with moderated or severe HIE. Enrolled cases abided by the following inclusion criteria: (1) all the neonates met the diagnostic criteria of hypoxic-ischemic encephalopathy formulated by the neonatal Group of the Pediatric Society of Chinese Medical Association; (2) all the neonates resided in the Neonatal Intensive Care Unit of Henan Children’s Hospital and had typical clinical manifestations, which were confirmed by computed tomography, magnetic resonance imaging and other examinations; (3) the demographic information and clinical characteristics of all neonates were complete. Exclusion criteria were as follows: (1) severe cardiac, liver and kidney dysfunction at birth; (2) neonates with hematological or congenital diseases; (3) neonates with convulsions caused by childbirth injury and intracranial hemorrhage.

### Data sources

Basic demographic information and clinical characteristics were derived from the neonatal HIE database. The neonatal HIE database in Henan Children’s Hospital is a computerized, disease-specific tool used for clinical applications. Data on maternal and neonatal characteristics of mild HIE and moderate or severe HIE groups were collected, consisting of (1) neonatal variables: age at diagnosis, gender, residential region, birth weight, gestational age, feeding pattern, 1-minute Apgar score, and 5-minute Apgar score; (2) maternal variables: parity, placenta abnormality, intrauterine infection, delivery mode, abnormal labor stage and amniotic fluid contamination. All of the information was categorized by trained specialists. In order to ensure the accuracy of the information, we regularly checked the items and logical errors in the database. When data were found to be inconsistent, the hospital number, recorded names, variables and errors would be listed to facilitate subsequent inspection and correction.

### Ascertainment of variables

In this study, neonatal birth weight was divided into low birth weight (1500–2499 g), normal birth weight (2500–4000 g) and macrosomia (>4000 g). According to neonatal gestational age, neonates were classified as premature infant (32–36 weeks), full-term infant (37–42 weeks) and postterm infant (>42 weeks). The feeding pattern was classified as breastfeeding, artificial feeding and mixed feeding. Abnormal labor stage referred to a total labor course ≤ 3 h or ≥ 24 h, a second stage of labor ≥ 2 h for primipara and ≥ 1 h for pluripara. Delivery mode was categorized into natural delivery and cesarean section. Cesarean section can improve infant or maternal outcomes only when used appropriately. In this study, we divided cesarean section into two groups: emergency cesarean section and planned cesarean section based on the reasons for its execution. Emergency cesarean section is a surgical procedure that is performed when there is an immediate threat to the life of fetus and/or woman. Placenta abnormality, intrauterine infection and amniotic fluid contamination were all classified as dichotomous variables consisting of “Yes” or “No”.

### Statistical analysis

The descriptive statistics were summarized using frequencies and percentages or mean ± standard deviations stratified by groups (moderate or severe HIE group vs. mild HIE group). T-test and chi-square test were applied to find associations or significant differences in the continuous variables and categorical variables, respectively, between the two groups. Since there were significant differences in the proportion and incidence of HIE between males and females, we conducted a gender-based stratified analysis to examine the distribution of potential influencing factors that affect HIE severity. The occurrence of moderate or severe HIE was taken as a dependent variable, and neonates with mild HIE were used as the reference group. Multiple logistic regression analysis was used to identify risk factors for predicting moderate or severe HIE stratified by gender. The meaningful variables of univariate analysis were included as candidate predictors in the multivariate model, and the stepwise method was used for model selection. Risks were estimated by unadjusted and adjusted odds ratios (ORs) and corresponding 95% confidence intervals (CI) of mild HIE vs. moderate or severe HIE. First, we estimated the unadjusted ORs in model 1. Subsequently, we adjusted ORs for birth weight, gestational age, parity, feeding pattern, 1-minute Apgar score, 5-minute Apgar score, placenta abnormality and intrauterine infection in model 2.

In addition, we also used an additive model to test for possible biological interactions between emergency cesarean section and either abnormal labor stage or amniotic fluid contamination. The relative excess risk due to interaction (RERI) and the attributable proportion due to interaction (AP) were evaluated to measure the additive interaction. When the 95% CI of RERI and AP were both excluded from zero, the additive interaction was considered significant. To estimate the multiplicative interaction, the product term was included in the logistic regression model and the interaction of odds ratio (IOR) and its 95% CI were calculated. If the 95% CI of the IOR does not include one, a significant multiplicative interaction was considered to exist. When the 95% CI of the IOR includes one, it was considered that there was no significant multiplicative interaction between the risk factors. All statistical analyses were performed using IBM software SPSS (version 21, Chicago, IL, USA). All calculated *P*-values were two-sided, and *P*-values less than 0.05 were considered statistically significant.

## Results

### Demographic characteristics of the study population

A total of 196 neonates diagnosed with HIE were enrolled in the final analysis, including 116 (59.2%) male and 80 (40.8%) female newborns. Among the study population, 39 (19.9%) neonates were diagnosed with mild HIE, 157 (80.1%) neonates were diagnosed with moderated or severe HIE. Maternal and neonatal related demographic characteristics of mild HIE and moderate or severe HIE were displayed in Table 1. The neonatal 1-minute Apgar score (2.73 ± 2.56 vs. 4.25 ± 2.97, *P* < 0.001) and 5-minute Apgar score (4.25 ± 2.97 vs. 5.68 ± 3.12, *P* < 0.001) were significantly lower in the moderate or severe HIE group compared to the mild HIE group. Maternal abnormal labor stage occurred in 73.6% of the moderate or severe HIE group compared to 26.4% in the mild HIE group (*P* = 0.013). The proportion of maternal amniotic fluid contamination in the moderate or severe HIE group (73.8%) was significantly higher compared to the mild HIE group (73.8% vs. 26.2%, *P* = 0.020). However, there were no statistically significant differences in the neonatal age at diagnosis, gender, residential region, feeding pattern, birth weight, gestational age, maternal parity, placenta abnormality and intrauterine infection between the two groups (all *P*>0.05).

**Table 1:** Maternal and neonatal demographic characteristics between the different levels of hypoxic ischemic encephalopathy (HIE)

Variables	Total (*n* = 196)	mild HIE (*n* = 39)	moderate or severe HIE (*n* = 157)	*P*-value
Age at diagnosis(days)	13.03 ± 5.13	12.33 ± 5.52	13.21 ± 5.27	0.346
Gender				0.288
Male	116(59.2)	26(66.7)	90(57.3)	
Female	80(40.8)	13(33.3)	67(42.7)	
Residential region				0.353
Urban	68(34.7)	16(41.0)	52(33.1)	
Rural	127(65.3)	23(59.0)	105(66.9)	
Birth weight(g)				0.944
1500–2499	101(51.5)	21(53.9)	80(51.0)	
2500–4000	38(19.4)	7(17.9)	31(19.7)	
>4000	57(29.1)	11(28.2)	46(29.3)	
Gestational age (weeks)				0.695
32–36	86(43.9)	18(46.2)	68(46.2)	
37–42	49(25.0)	11(28.2)	38(24.2)	
>42	61(31.1)	10(25.6)	51(25.6)	
Parity				0.156
1	112(57.2)	19(48.7)	93(59.3)	
2	61(31.1)	17(43.6)	44(28.0)	
≥ 3	23(11.7)	3(7.7)	20(12.7)	
Feeding pattern				0.260
Breastfeeding	92(46.9)	15(38.5)	77(49.1)	
Artificial feeding	78(39.8)	20(51.3)	58(36.9)	
Mixed feeding	26(13.3)	4(10.2)	22(14.0)	
1-minute Apgar score	6.16 ± 3.52	3.59 ± 2.33	2.73 ± 2.56	<0.001
5-minute Apgar score	6.81 ± 2.57	5.68 ± 3.12	4.25 ± 2.97	<0.001
Placenta abnormality				0.982
Yes	55(28.1)	11(28.2)	44(28.0)	
No	141(71.9)	28(71.8)	113(72.0)	
Intrauterine infection				0.298
Yes	110(56.1)	19(48.7)	91(58.0)	
No	86(43.9)	20(51.3)	66(42.0)	
Delivery mode				0.003
Natural delivery	69(35.2)	23(59.0)	46(29.3)	
Emergency cesarean section	85(43.4)	9(23.1)	86(48.4)	
Planned cesarean section	42(21.4)	7(17.9)	35(22.3)	
Abnormal labor stage				0.013
Yes	90(45.9)	11(28.2)	79(50.3)	
No	106(54.1)	28(71.8)	78(49.7)	
Amniotic fluid contamination				0.020
Yes	93(47.4)	12(30.8)	81(51.6)	
No	103(52.6)	27(69.2)	76(48.4)	

### Subgroup analysis of potential factors influencing neonatal hypoxic ischemic encephalopathy based on gender

To investigate the distribution of potential factors that can influence neonatal HIE based on a subgroup analysis, neonates were divided into different gender groups. The frequencies of potential influencing factors among HIE patients based on gender are shown in Table 2. We found that only male neonates show significant differences in the frequence of emergency cesarean section, abnormal labor stage and amniotic fluid contamination between the mild and moderate or severe HIE groups (all *P*<0.05). There were no significant differences between the two groups based on birth weight (*P* = 0.658) or gestational age (*P* = 0.095) among male neonates. In the female neonates, there were no statistically significant differences detected between the two groups with respect to birth weight, gestational age, emergency cesarean section, abnormal labor stage and amniotic fluid contamination (all *P*>0.05).

**Table 2:** Frequence of potential factors influencing hypoxic ischemic encephalopathy (HIE) based on genders

Variables	Male				Female			
	Total	mild HIE	moderate or severe HIE	*P*-value	Total	mild HIE	moderate or severe HIE	*P*-value
Birth weight(g)				0.658				0.587
1500-2499	61(52.6)	14(53.9)	47(52.2)		40(50.0)	7(53.8)	33(49.3)	
2500-4000	17(14.6)	5(19.2)	12(13.3)		21(26.2)	2(15.4)	19(28.4)	
＞4000	38(32.8)	7(26.9)	31(34.5)		19(23.8)	4(30.8)	15(22.3)	
Gestational age (weeks)				0.095				0.266
32-36	39(33.6)	11(42.3)	28(31.1)		47(58.7)	7(53.8)	40(59.7)	
37-42	29(25.0)	9(34.6)	20(22.2)		20(25.0)	2(15.4)	18(26.9)	
＞42	48(41.4)	6(23.1)	42(46.7)		13(16.3)	4(30.8)	9(13.4)	
Delivery mode				0.011				0.136
Natural delivery	39(33.6)	16(61.5)	23(25.6)		30(37.5)	7(53.8)	23(34.3)	
Emergency cesarean section	52(44.8)	6(23.1)	46(51.1)		33(41.3)	4(30.8)	29(43.3)	
Planned cesarean section	25(21.6)	4(15.4)	21(23.3)		17(21.2)	2(15.4)	15(22.4)	
Abnormal labor stage				0.008				0.325
Yes	62(53.4)	8(30.8)	54(60.0)		28(35.0)	3(23.1)	25(37.3)	
No	54(46.6)	18(69.2)	36(40.0)		52(65.0)	10(76.9)	42(62.7)	
Amniotic fluid contamination				0.029				0.205
Yes	62(53.4)	9(34.6)	53(58.9)		31(38.8)	3(23.1)	28(41.8)	
No	54(46.6)	17(65.4)	37(41.1)		49(61.2)	10(76.9)	39(58.2)	

### Logistic regression analysis of the risk factors associated with moderate or severe hypoxic ischemic encephalopathy stratified by gender

Results for the logistic regression analysis of the risk factors associated with moderate or severe HIE according to gender are presented in Table 3. The unadjusted and adjusted ORs and 95%CI were reported in model 1 and model 2, respectively. Based on the multivariate logistic regression analysis, results showed that associations differed based on the gender. In the male neonates group, we found that emergency cesarean section, abnormal labor stage and amniotic fluid contamination were significantly associated with an increased risk of moderate or severe HIE compared to the mild HIE, where the adjusted ORs were 4.378(95%CI:2.263–6.382), 2.827 (95%CI:1.743–5.196) and 2.653 (95%CI:1.645–3.972), respectively. In the female neonates group, we also found that emergency cesarean section, abnormal labor stage and amniotic fluid contamination had a positively higher risk of moderate or severe HIE (adjusted ORs:4.211, 2.756 and 2.527; 95%CI:2.196–5.178, 1.817–4.985 and 1.496–4.013, respectively).

**Table 3:** Risk factors for moderate or severe hypoxic ischemic encephalopathy based on logistic regression analysis according to different gender

Variables	Model 1^a^		Model 2^b^	
	Unadjusted OR(95%CI)	*P*-value	Adjusted OR(95%CI)	*P*-value
Male				
Delivery mode		0.003		0.001
Natural delivery	1.00(reference)^c^		1.00(reference)^c^	
Emergency cesarean section	3.237(2.125-5.036)		4.378(2.263-6.382)	
Planned cesarean section	1.761(0.623-2.975)		2.256(0.897-3.362)	
Abnormal labor stage		0.017		0.021
Yes	2.356(1.316-4.235)		2.827(1.743-5.196)	
No	1.00(reference)^c^		1.00(reference)^c^	
Amniotic fluid contamination		0.025		0.027
Yes	2.175(1.568-3.736)		2.653(1.645-3.972)	
No	1.00(reference)^c^		1.00(reference)^c^	
Female				
Delivery mode		0.021		0.018
Natural delivery	1.00(reference)^c^		1.00(reference)^c^	
Emergency cesarean section	3.151(1.897-4.675)		4.211(2.196-5.178)	
Planned cesarean section	1.693(0.758-2.896)		2.109(0.935-3.177)	
Abnormal labor stage		0.024		0.035
Yes	2.243(1.565-4.297)		2.756(1.817-4.985)	
No	1.00(reference)^c^		1.00(reference)^c^	
Amniotic fluid contamination		0.038		0.042
Yes	2.098(1.275-3.876)		2.527(1.496-4.013)	
No	1.00(reference)^c^		1.00(reference)^c^	

^a^Model 1: the unadjusted ORs

^b^Model 2: adjusted ORs for the birth weight, gestational age, parity, feeding pattern, 1-minute Apgar score, 5-minute Apgar score, placenta abnormality and intrauterine infection

^c^1.00(reference) meant the reference group

### Interaction analysis between emergency cesarean section, abnormal labor stage, and amniotic fluid contamination

The results of interaction analysis between emergency cesarean section and abnormal labor stage, or amniotic fluid contamination are showed in Table 4. We found that patients in the moderate or severe HIE group with abnormal labor stage had significantly higher risk (OR = 4.352, 95%CI = 2.198–6.526), when comparing to the mild HIE group with abnormal labor stage. As expected, a significant additive effect was found when both emergency cesarean section and abnormal labor stage have occurred (RERI = 2.135, 95% CI = 1.573–3.652; AP = 0.716, 95%CI = 0.496–1.137). In addition, we also found that amniotic fluid contamination significantly increased the risk of moderate or severe HIE (OR = 3.971, 95%CI = 1.329–5.693) compared to mild HIE. Similarly, the occurrence of emergency cesarean section and amniotic fluid contamination also yielded cumulative effects (RERI = 1.896, 95% CI = 1.337–3.861; AP = 0.612, 95%CI = 0.853–1.665).

**Table 4:** The interaction analysis between emergency cesarean section and either abnormal labor stage or amniotic fluid contamination

Variables	Emergency cesarean section	Adjusted OR(95%CI)	IOR(95%CI)	RERI(95%CI)	AP(95%CI)
Abnormal labor stage			3.258 (1.173–5.865)	2.315 (1.573–3.652)	0.716 (0.496–1.137)
Yes	moderate or severe HIE	4.352 (2.198–6.526)			
No	mild HIE	1.00(reference)^a^			
Amniotic fluid contamination			2.231 (1.549–3.769)	1.896 (1.337–3.861)	0.612 (0.853–1.665)
Yes	moderate or severe HIE	1.00(reference)^a^			
No	mild HIE	3.971 (1.329–5.693)			

^a^1.00(reference) meant the reference group

## Discussion

Multiple factors can contribute to the development of moderate or severe HIE throughout the neonatal period. In this cross-sectional study, we examined various factors between moderate or severe HIE and mild HIE and conducted a subgroup analysis by gender. The logistic regression analysis results showed that neonates with emergency cesarean section, abnormal labor stage and amniotic fluid contamination had an increased risk of moderate or severe HIE. In addition, we also found cumulative effects upon the concurrence of emergency cesarean section and abnormal labor stage, as well as emergency cesarean section and amniotic fluid contamination. Among the neonatal factors, there was a statistically significant relationship between moderate or severe HIE and male gender. Our study showed that there was higher male susceptibility to neonatal moderate or severe HIE, which suggests that one of the risk factors is gender. It has been reported that male neonates tend to have unfavorable outcomes of moderate or severe HIE compared to female ones, which has been demonstrated using radiological methods such as magnetic resonance imaging and ultrasound [22, 23].

With regard to the delivery mode, our study showed that emergency cesarean section in male neonates (adjusted OR:4.378, 95%CI:2.263–6.382) and female neonates (adjusted OR:4.211, 95%CI:2.196–5.178) were all associated with an increased risk of moderate or severe HIE. These findings were consistent with previous studies. In a Swedish national population-based cohort study, Liljestrom et al. [24] revealed that cesarean delivery was a major antepartum risk factor for moderate to severe hypoxic ischemic encephalopathy. In a case-control study in Southern China [25], the multivariate logistic regression analysis confirmed that cesarean section can increase the risk of HIE(OR:3.924, 95%CI:2.046–5.875). Therefore, emergency cesarean section is not protective for moderate or severe HIE. One speculated reason for the increased risk is that emergency cesarean section may have been performed too late in cases of moderate or severe HIE, leading to brain hypoxia. Inappropriate management of delivery or delayed alertness in the emergency operative room can lead to serious cases of delayed cesarean section. In general, natural delivery is considered safer than cesarean section, both in the short and long term. In order to prevent delayed delivery caused neonatal cerebral hypoxia in the process of delivery, clinicians must make the important choice of delivery mode after appropriate risk assessment and attempt to shorten or complete the process of delivery as soon as possible.

In this study, abnormal labor stage was an independent risk factor for moderate or severe HIE in male neonates (adjusted OR:2.827, 95%CI:1.743–5.196) and female neonates (adjusted OR:2.756, 95%CI:1.817–4.985), which is consistent with other recent studies that demonstrated an association between abnormal labor stage of delivery and adverse neonatal outcomes. A case-control study was conducted in the Neonatal Unit of the Children’s Hospital and the Institute of Child Health, Lahore, Pakistan, where they found possible associations between abnormal labor stage and HIE in 153 neonatal HIE cases compared to 187 controls (OR:6.3, 95% CI:3.3–11.9) [26]. Torbenson et al. performed a case-controlled study, which found that abnormal labor stage (adjusted OR:9.5, 95% CI:1.0-135.3) was significantly associated with HIE [27]. Interestingly, we also found an additive effect upon the interaction of emergency cesarean section and abnormal labor stage in our study. The association of abnormal labor stage and moderate or severe HIE may be multifactorial. One reason for this may be that moderate or severe cases of HIE may have had emergency cesarean section complicated by hypoxia, which is more likely to occur after abnormal labor stage. Long-term hypoxia can cause acidosis, inhibit respiratory center and lead to neonatal moderate or severe HIE. Therefore, this result shows that it is necessary to improve awareness when monitoring the delivery process, so that clinicians can take corresponding treatment measures to reduce the incidence of asphyxia caused by the abnormal labor stage, which can lead to moderate or severe HIE.

A significant correlation between amniotic fluid contamination and moderate or severe HIE has been observed in this study. Amniotic fluid contamination is generally recognized as a risk factor of concern. Our study showed that after adjusting for potential confounders, male and female neonates with amniotic fluid contamination were 2.653 times and 2.527 times more likely to develop moderate or severe HIE compared to controls, respectively. This finding was consistent with previous studies. Chen X et al. [28] found that amniotic fluid contamination (OR:3.223, 95% CI:1.049–9.901) may increase the risk of HIE. Wang J et al. [25] also concluded that amniotic fluid contamination was associated with an increased risk of HIE (OR:4.527,95%CI: 2.704–5.483) in a case-control study conducted in Southern China. Above all, the incidence of moderate or severe HIE increases when emergency cesarean section and amniotic fluid contamination interactions occur. The significant additive effect and multiplicative effect in the interactions were observed between emergency cesarean section and amniotic fluid contamination. Cesarean section, especially elective emergency cesarean section before labor, is prone to fluid retention in the lungs, resulting in an increased risk of asphyxia after birth. Sometimes the newborn is in good condition at birth and the Apgar score is not low, but then the score drops rapidly and turns into a state of asphyxia, which makes rescue difficult. Neonates with intrauterine hypoxia, hyperperistalsis, and analsphincter relaxation can lead to meconium discharge contaminating the amniotic fluid. At present, amniotic fluid contamination during pregnancy is an important clinical factor leading to fetal intrauterine hypoxia or distress, and the degree of amniotic fluid contamination is directly correlated with neonatal asphyxia. Thus, clinicians should promptly evaluate the fetal status and have a full understanding of the growth and development of neonates [29], which includes being familiar with the condition of the amniotic fluid. Once the neonate is in asphyxia or distress, the correct countermeasures need to be taken to end of delivery in a timely manner if necessary.

Clinically, it is not only necessary to take appropriate preventive and therapeutic measures, but also to identify the relevant risk factors leading to the incidence of moderate or severe HIE so that early intervention strategies and tailored therapies can be applied before the severity of the disease exacerbates. Identifying these clinical risk factors of moderate or severe HIE is important to help clinicians to strengthen the relevant awareness, and also for the centralization of births in second level centers in order to minimize adverse outcomes [30–37]. At present, the risks of moderate or severe HIE are mainly based on the characteristics of pregnant mother and the fetus during pregnancy and delivery. Maternal and neonatal healthcare indicators are internationally recognized as the best to evaluate the quality of health care in a country. A 2-year period study conducted in Sicily (Italy) [38], found that in most cases (81%) there was a correlation between obstetric complications/non-physiological pregnancies and newborns transferred to II level centers. This highlights the continuity of care between maternal and newborn, as well as the unpredictability of birth, whose complications are not prevented in all cases excluding maternal/fetal risk factors. Moreover, evidence-based medical research shows that hypothermia therapy is one of the main measures for the treatment of neonatal moderate or severe HIE at present, which can effectively reduce the disability and mortality rates of neonates, improve their neurological function and promote their intellectual development. Therefore, the quality of adequate obstetric and neonatal management, oriented towards neurocritical care, also in other settings and especially considering the therapeutic perspectives provided by hypothermia is critical for prevent perinatal mortality/morbidity. Clinicians must be realized to keep pace with the times, and to guarantee a careful and updated clinical care. Based on the present analysis, we believe that improvements in the observed critical areas may have a positive impact on maternal and newborn health outcomes, and avoid many of maternal/fetal/neonatal deaths and diseases. The provision of humanized and high-quality care is therefore likely to have beneficial short- and long-term effects on maternal and neonatal health [39, 40].

The main strengths of this study include the relatively large number of neonates enrolled, which allowed for the analysis of several potential confounding factors, as well as a gender stratified analysis. All cases came from a single hospital institution and the variables were collected in a uniform manner, thus minimizing differences in outcome assessment and limiting information bias. In addition, we used neonates with mild HIE as controls, which allowed us to focus on a population of moderate or severe neonates with HIE, who have significant morbidity and who would benefit the most from HIE prevention. There are several limitations that should be considered. First, given that the study was conducted in a cross-sectional design, the causal pathways underlying the observed relationships are difficult to verify. If permitted, further cohort studies with large sample sizes are needed. Second, our findings mainly focused on exploring the risk factors of neonatal moderate or severe HIE, and potential variables affecting HIE may not have shown statistical significance. Third, though our present study included a large sample from Henan province, the findings may not be universally applicable to populations with different demographic, socioeconomic or healthcare system characteristics. Therefore, our findings need to be replicated via an in-depth study covering a wider range of different characteristics sample populations in the future.

## Conclusions

We found that emergency cesarean section, abnormal labor stage, and amniotic fluid contamination were risk factors for moderate or severe HIE. There was a statistically significant relationship between moderate or severe HIE and male gender, suggesting that sex should be regarded as a specific stratified characteristic. Notably, there were statistically significant interactions between emergency cesarean section and abnormal labor stage, and between emergency cesarean section and amniotic fluid contamination. It is necessary for clinicians to pay attention to these risk factors, in order to strengthen their awareness in clinical practice and to reduce the incidence of moderate or severe HIE in the early neonatal period.

## Data Availability

The datasets used and/or analysed during the current study are available from the corresponding author on reasonable request.

## Abbreviations

ORs: Odds Ratios
CIs: Confidence Intervals
RERI: relative excess risk due to interaction
AP: attributable proportion due to interaction
IOR: Interaction of Odd Ratio
